# An Unusual Friedel–Crafts Reaction and Violation of the Markovnikov Rule in the Formation of an Adamantyl Arene

**DOI:** 10.1021/acs.joc.5c00215

**Published:** 2025-04-14

**Authors:** B. Andes Hess, Lidia Smentek

**Affiliations:** Department of Chemistry, Vanderbilt University, Nashville, Tennessee 37235, United States

## Abstract

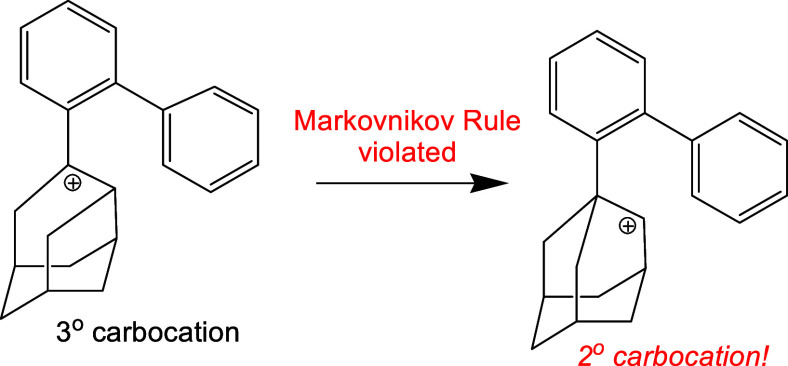

Yoshihara, Hiroki, Yagi, and Itami utilized an unusual Friedel–Crafts-like alkylation in the synthesis of an adamantane-annulated arene. The precursor was a 3° carbocation, which they proposed undergoes a Wagner–Meerwein shift to a 2° carbocation, which subsequently alkylates a benzene ring. This mechanism was supported by their DFT calculations. However, their choice of the B3LYP functional often finds false transition structures. A more appropriate functional showed that the alkylation occurs via a concerted reaction.

## Introduction

When the Markovnikov Rule was published,^1^ it was applied to additions of HX to unsymmetrical C–C double bonds. Subsequently with the discovery of the electron and the importance of charges, the rule was restated that the positively charged ion (e.g., H^+^) would add to the unsymmetrical double bond to form the more stable carbocation. With the discovery of Wagner–Meerwein rearrangements of carbocations in 1922^2^ the rule was eventually applied to predict the more stable carbocation formation.

**Scheme 1:**
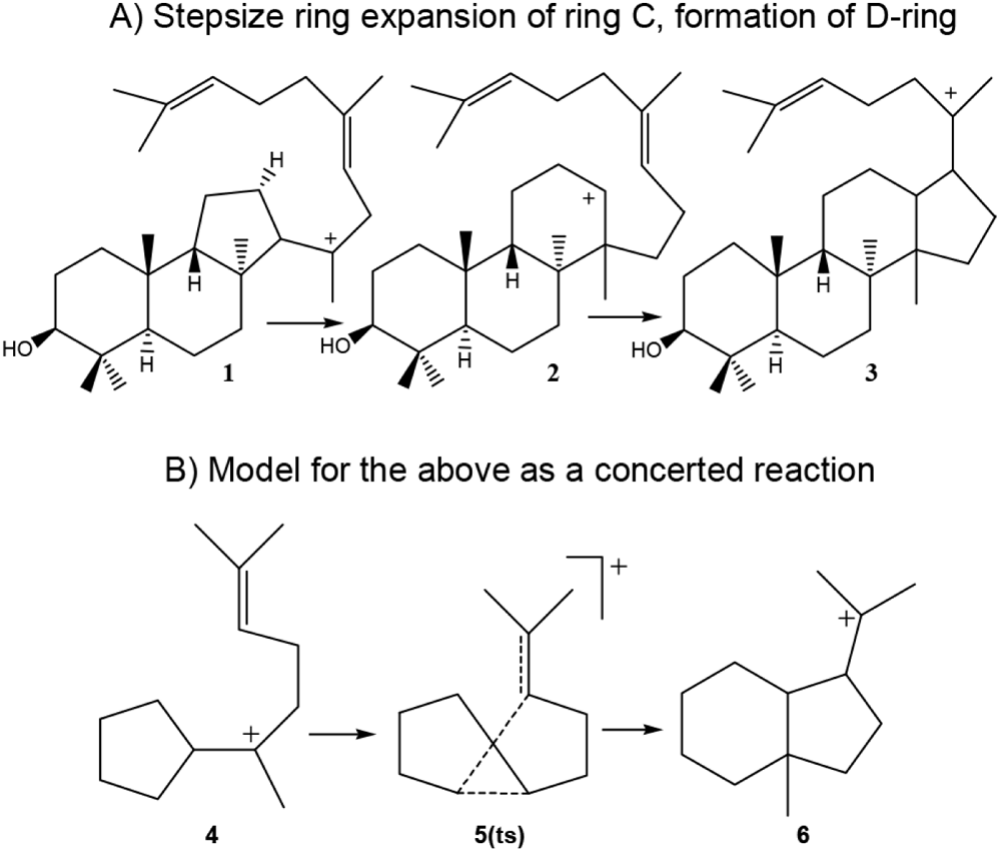
Proposed Formation of C and D Rings in Biosynthesis of Lanosterol

The question of the violation of the Markovnikov Rule first became apparent in Wagner–Meerwein shifts in the study of biochemical syntheses. Corey and Matsuda concluded in 1997 that indeed, an anti-Markovnikov formation occurred^3^ in the conversion of **2** from **1** in Scheme 1. By the year 2000 introductory textbooks in organic chemistry showed this anti-Markovnikov ring expansion (Scheme 1A). In 2002 it was proposed (Scheme 1B) that there might be a way to avoid the formation of a secondary carbocation in the formation of **3** using a simple model in which **4** undergoes a concerted reaction to give **6** via transition structure **5(ts)**.^4^ Subsequent computational studies on the overall conversion of squalene oxide to the protosterol cation (**3**) confirmed that this anti-Markovnikov ring expansion was avoided. A concerted ring expansion of ring C and formation of pentacyclic ring D occurred in both the gas phase and in the presence of an enzyme.^5^

In the past two decades there have been many studies published on the biosynthesis of a wide variety of terpenes. In the majority of cases the appearance of secondary carbocations was avoided by a concerted reaction^6^ in a very similar way to the model system of a concerted reaction reported in 2002.^4^

## Results and Discussion

Recently Yagi and Itami^7^ have reported a similar type of reaction in which a cyclization occurs with a carbocation. In this case however the cyclization involves a Friedel–Crafts alkylation that is very different from the Wagner–Meerwein shift observed in the biosynthesis in the above terpenes. Namely, a carbocation is involved in a “classic” Friedel–Crafts alkylation. One might expect that the carbocation would be too weak as an electrophile to break the aromaticity in the electrophilic substitution.

There are many examples of enzymatically catalyzed Friedel–Crafts reactions. As seen from three recent reviews of these reactions^8^ there is no example of a nonactivated benzene ring undergoing an enzyme catalyzed Friedel–Crafts reaction. It is well-known that the lone pairs of electrons on oxygen can increase the reactivity of a benzene ring, since they lower the activation energy toward electrophilic attack by the electrophile. In collaboration with Piel, we have recently carried out a detailed DFT computational study on sterol-like cyclization of a monodomain Class II terpene cyclase.^9^ The benzene was highly activated toward electrophilic substitution by the presence of two OH substituents.

**Figure 1:**
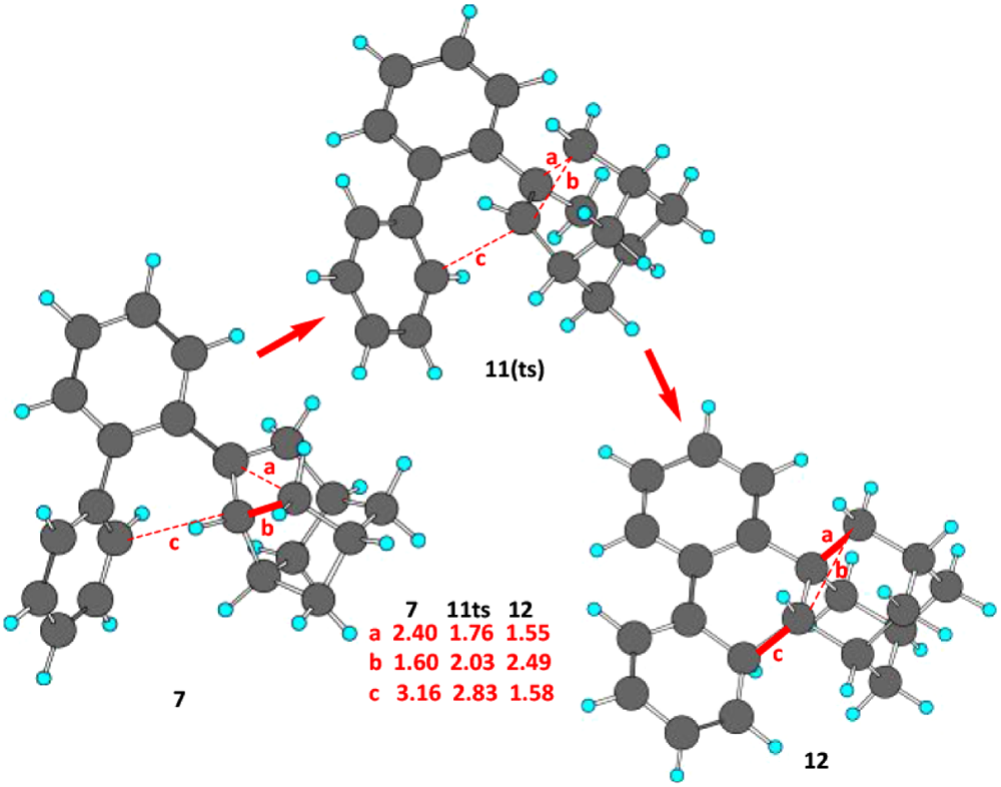
Conversion of the tertiary carbocation **7** via the concerted transition structure 11(ts) to the aromatic, electrophilic substitution intermediate **12**, which loses H^+^ to give **10**. The solid bonds designate the breaking bond in **7** and newly formed bonds in **12**.

To our knowledge, there is no example of a carbocation undergoing Friedel–Crafts alkylation without the benzene ring being activated by a strongly electron donating substituent, such as a hydroxy or amino group. This is best seen by their Hammett sigma constants having large negative values.^10^ In this case the only substituent on the benzene ring being attacked by the electrophilic carbocation is the phenyl group, which has a sigma constant of −0.01. Furthermore, the phenyl group is significantly out of the plane of the benzene ring being attacked (Figure 1), lessening the conjugation between the two rings.

However, a referee drew our attention to an intramolecular Friedel–Crafts reaction in which a benzene ring is attacked by a carbocation that has only one substituent, a cyclopropane ring.^11^ It is well-known that a cyclopropane does conjugate with an adjacent sp^2^ hybridized carbon, which might assist in this aromatic substitution reaaction.^12^

**Scheme 2:**
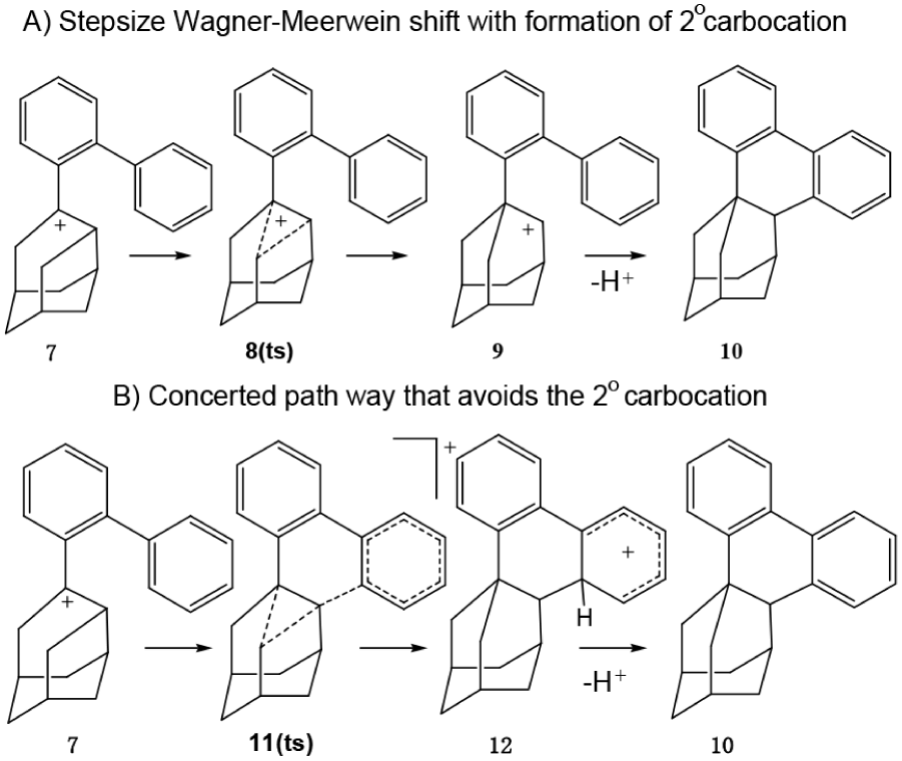
Proposed Mechanisms for Formation of **10**

**Figure 2:**
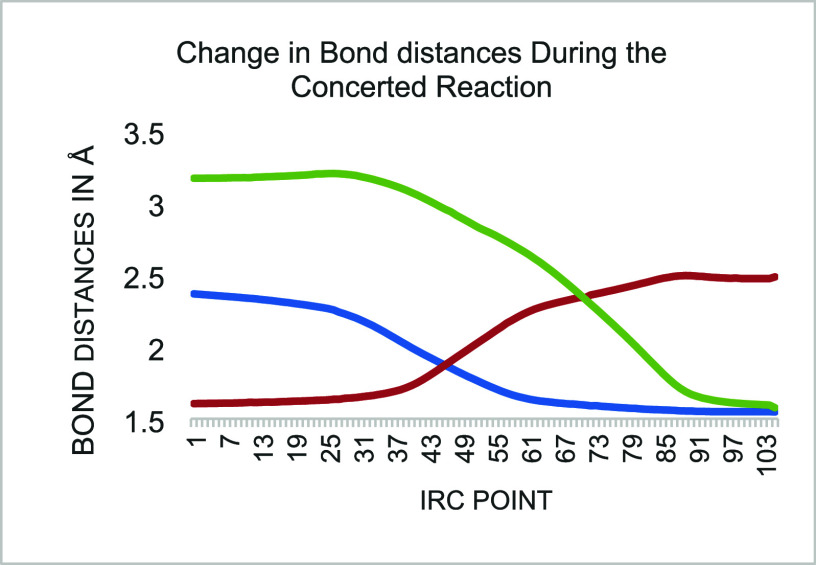
Intrinsic reaction coordinate of the concerted reaction. Green line is bond c, blue line bond a, red line bond b.

**Figure 3:**
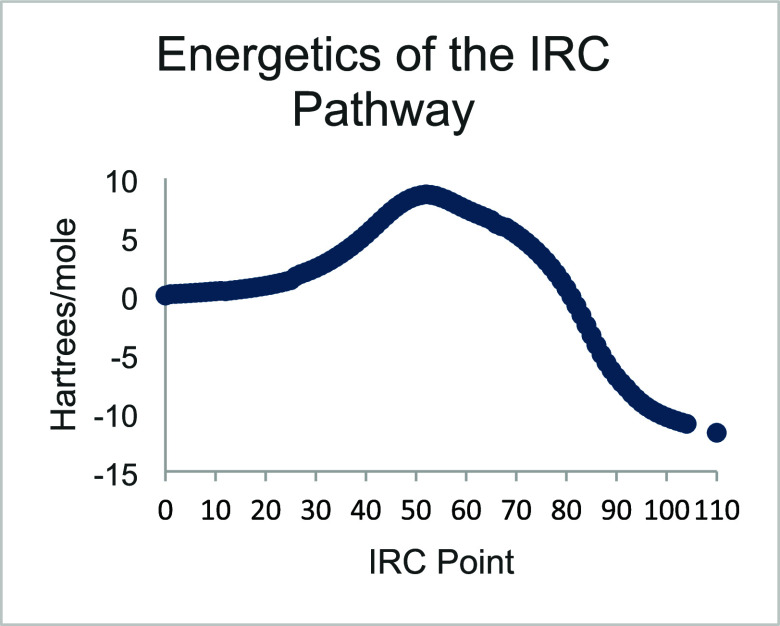
Changes in the potential energy during the course of the IRC pathway.

Before further consideration of this Friedel–Crafts reaction, let us first consider the authors’ proposed presence of a secondary carbocation **9** in Scheme 2A. As mentioned earlier there are many known cases of nature avoiding forming a secondary carbocation from a tertiary carbocation. The authors used the B3LYP functional for their density functional theory (DFT) calculations (Scheme 2A). Unfortunately, it is well-known that this functional can provide spurious results in calculating the potential surface of a reaction. This is especially the case when transition structures are found not to exist when a better functional such as M06-2X^13^ is used. Therefore, we performed the computation of the potential surface for the conversion of **7** to **12** with the M06–2X functional^14^ with the 6–31G* basis set.^15^ Not surprisingly as shown in Scheme 2B, the secondary carbocation was not found, but instead a concerted reaction occurred for the conversion **7** to **12** with the transition structure **11ts** linking them. In Figure 1 the Chem3D structures are shown in which the bonds are formed and those broken are given. An intrinsic reaction coordinate (IRC) diagram (Figure 2) shows the smooth change in bond distances of the three key bonds (a, b and c). The energetics of the IRC pathway are shown as the reaction proceeds in Figure 3.

**Scheme 3:**
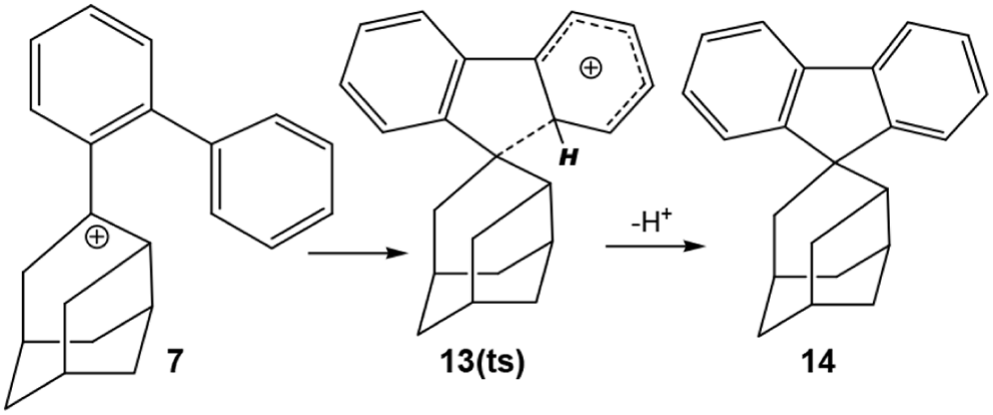
Formation of the Spiro Product **14**

The authors also considered a second reaction pathway of **7** in which a spiro system (**14**) is formed via transition structure **13(ts)**, Scheme 3, though they did not isolate any of the spiro products.

**Figure 4:**
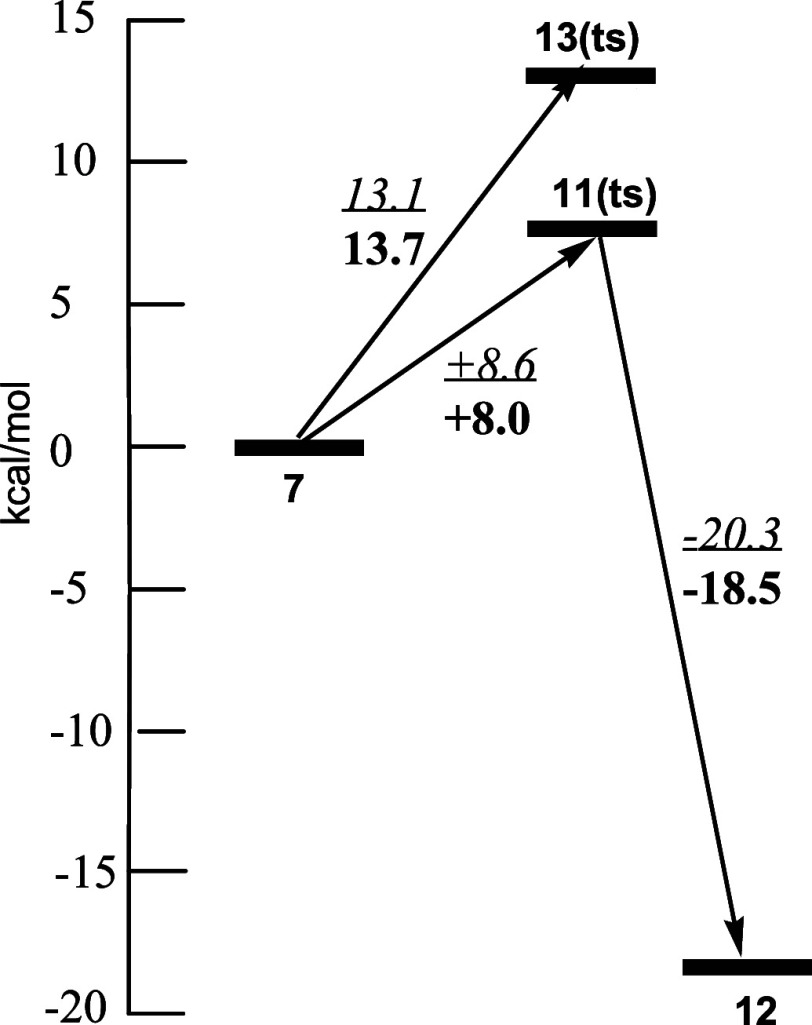
Relative energies of the stationary points of the two potential energy pathways. The numbers in italics are with the 6-31G* and in boldface with 6-31+(d,p) basis set.

The relative energies of the components of these two pathways are given in Figure 4. Given the difference in activation energies of the two pathways, it is not surprising that no spiro product (**14**) was isolated.

## Conclusion

**Scheme 4:**
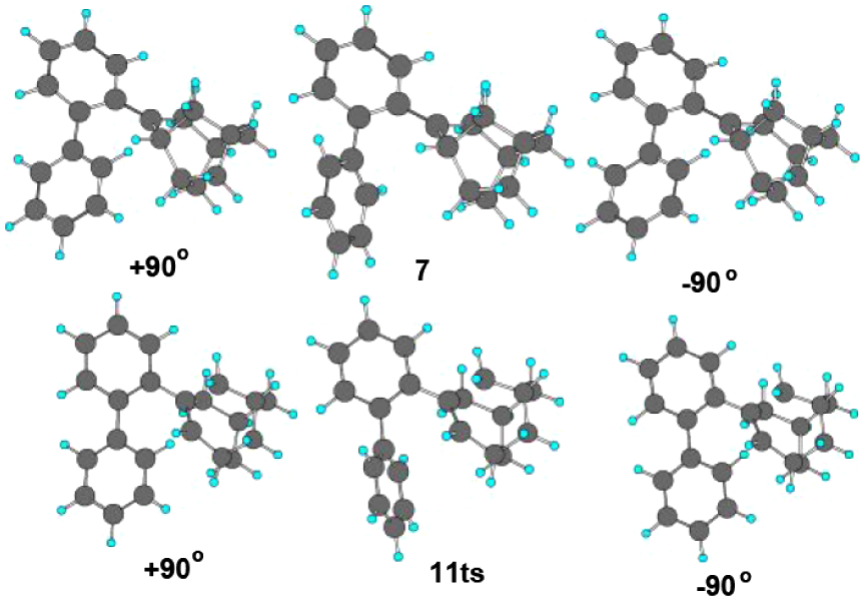
Conformational Analysis of **7** and **11ts** Showing Rotation about the C–C Bond Linking the Two Benzene Rings

Finally, one must ask the question what is so special about this adamantyl arene system that allows a Friedel–Crafts alkylation to occur between a carbocation and a benzene ring with no electron donating substituents on the benzene ring. A conformational analysis (Scheme 4) of a model shows that the carbocation is locked into a conformation due to steric hindrance that is favorable for its electrophilic attack on the benzene ring. The intramolecular nature of this reaction might also be a reason that a Friedel–Crafts reaction occurs in this system.

## Data Availability

The data underlying this study are available in the published article and its Supporting Information.

## Supporting Information Available

Computational results, energies, and Cartesian coordinates of all optimized structures located in this work (PDF)
