# Supplementary material for: An Unusual Friedel–Crafts Reaction and Violation of the Markovnikov Rule in the Formation of an Adamantyl Arene
Source: J Org Chem. 2025 Apr 14;90(16):5546–9. doi: 10.1021/acs.joc.5c00215 (PMC12038835; doi:10.1021/acs.joc.5c00215)
Supplement: Supplementary file 1 — jo5c00215_si_001.pdf [file jo5c00215_si_001.pdf]

Supporting Information

An Unusual Friedel-Crafts Reaction and Violation of the Markovnikov Rule in the Formation of an Adamantyl Arene

B. Andes Hess, Jr.* and Lidia Smentek

Department of Chemistry, Vanderbilt University, Nashville, TN 37235, United States

Computational Details.

Computational results for all structures for which structures were computed with the density functional method using the M062X functional and 6-31G* and 6-31+G(d,p) basis sets are given below.

7

#M062X/6-31G(d) freq(noraman) nopop guess=read geom=check

1	6	0	3.161469	0.302952	-0.076860
2	6	0	3.694168	-0.053247	1.326285
3	6	0	2.723914	-0.918272	2.145734
4	6	0	2.370396	-2.190858	1.370922
5	6	0	1.597884	-1.607019	0.178527
6	6	0	2.596020	-0.941726	-0.775174
7	6	0	1.348433	-0.255670	2.266207
8	6	0	0.668523	-0.528890	0.845096
9	6	0	0.683108	0.796265	0.211569
10	6	0	-0.491399	1.581293	0.009809
11	6	0	-1.771936	1.003395	-0.261419
12	6	0	-2.899620	1.819496	-0.183420
13	6	0	-2.785259	3.175585	0.102927
14	6	0	-1.528790	3.767764	0.281037
15	6	0	-0.395716	2.985482	0.211802
16	6	0	-1.906335	-0.368541	-0.789200
17	6	0	-1.042689	-0.812284	-1.801529
18	6	0	-1.179706	-2.090657	-2.333231
19	6	0	-2.175100	-2.938976	-1.854862
20	6	0	-3.044368	-2.501323	-0.854938
21	6	0	-2.921181	-1.219814	-0.332242
22	6	0	2.043571	1.366129	-0.064078
23	1	0	3.987271	0.723372	-0.658868
24	1	0	3.937080	0.865631	1.873812
25	1	0	4.634214	-0.604462	1.207121
26	1	0	3.148049	-1.121146	3.133867
27	1	0	1.735031	-2.847045	1.975398
28	1	0	3.245524	-2.765034	1.051544
29	1	0	0.975710	-2.337792	-0.344429
30	1	0	2.114058	-0.656367	-1.720108
31	1	0	3.393527	-1.649411	-1.021999
32	1	0	1.389445	0.802446	2.544236
33	1	0	0.722476	-0.759779	3.007503
34	1	0	-0.342302	-0.902332	0.990685
35	1	0	-3.872090	1.395666	-0.413757
36	1	0	-3.680123	3.788546	0.150385
37	1	0	-1.447196	4.830678	0.478765

38	1	0	0.572852	3.439179	0.389869
39	1	0	-0.297165	-0.132060	-2.208121
40	1	0	-0.519314	-2.417248	-3.130364
41	1	0	-2.284217	-3.935883	-2.269166
42	1	0	-3.821862	-3.160908	-0.483794
43	1	0	-3.591541	-0.884817	0.454566
44	1	0	2.264442	2.145783	0.680036
45	1	0	1.992730	1.888446	-1.027924

SCF Done: E(RM062X) = -851.582526253 A.U. after 1 cycles
Frequencies -- 10.9749 48.9689 66.7144
Zero-point correction= 0.397230 (Hartree/Particle)
Thermal correction to Energy= 0.413777
Thermal correction to Enthalpy= 0.414721
Thermal correction to Gibbs Free Energy= 0.352020
Sum of electronic and zero-point Energies= -851.185297
Sum of electronic and thermal Energies= -851.168749
Sum of electronic and thermal Enthalpies= -851.167805
Sum of electronic and thermal Free Energies= -851.230506
Maximum Force 0.000005 0.000450 YES
RMS Force 0.000001 0.000300 YES
Maximum Displacement 0.001571 0.001800 YES
RMS Displacement 0.000343 0.001200 YES

11(TS)

#M062X/6-31G(d) freq(noraman) nopop guess=read geom=check

C 3.2604312522,-0.7396216727,-0.373332481
C 3.8106103065,-0.7284352882,1.0609312258
C 2.762792517,-1.2768101381,2.0418940358
C 2.4148888889,-2.7241349862,1.6884202081
C 1.7505837094,-2.6283130652,0.280154892
C 2.7814691183,-2.1492166497,-0.7516717473
C 1.4675775307,-0.4741412283,1.9622472222
C 0.674605475,-1.6225275063,0.4847411706
C 0.8743631591,-0.2402184186,0.3225133752
C -0.3390840576,0.6491872186,0.3027431528
C -1.5369113034,0.2130044735,-0.3026484776
C -2.636941507,1.0764210191,-0.3031164761
C -2.5594554887,2.3458680387,0.2579886806
C -1.3660744748,2.7825443244,0.8256723853
C -0.2651807255,1.9353187297,0.8420865012
C -1.6690881828,-1.1254525256,-0.9447451019
C -0.7739107214,-1.542714149,-1.9409151607
C -0.8689598433,-2.8188601331,-2.4928150034
C -1.8632876804,-3.6928652941,-2.0594529164
C -2.7728515085,-3.278435154,-1.0876993347
C -2.6780410519,-2.0042506969,-0.5343756088
C 2.0802006865,0.2301956293,-0.4875856435
H 4.0436529489,-0.4173071809,-1.065937181
H 4.0994468907,0.2904346327,1.3443526209
H 4.7128729306,-1.3464480825,1.1242225626
H 3.1335668468,-1.2058593417,3.0711048053
H 1.7204541539,-3.1554077404,2.4170336482
H 3.2965908906,-3.3698287356,1.6357766444
H 1.2926381388,-3.5847985109,0.0093784099
H 2.334864143,-2.1463450739,-1.7526673501
H 3.6199398321,-2.8528636007,-0.7707522151

H 1.6113620296,0.5971537386,2.1301273859
H 0.6851139956,-0.8079370456,2.6486201189
H -0.2691727455,-1.9547617263,0.9132144078
H -3.5569941442,0.7452255278,-0.7756560405
H -3.4263758227,2.9980219949,0.2405366207
H -1.291650986,3.7757587155,1.2550241391
H 0.6615028506,2.2872786498,1.2905098582
H -0.0257371774,-0.8463820322,-2.3133122129
H -0.1795719787,-3.120575319,-3.2756698941
H -1.9409023007,-4.6861031909,-2.48928378
H -3.5579291974,-3.9508745389,-0.7570906821
H -3.3788804287,-1.6901767197,0.2345845661
H 2.3577762035,1.2373728718,-0.1564071822
H 1.7486508284,0.3295891808,-1.5280861483
SCF Done: E(RM062X) = -851.568773047 A.U. after 1 cycles
Frequencies -- -210.1456 52.6712 57.9990
Zero-point correction= 0.396867 (Hartree/Particle)
Thermal correction to Energy= 0.412745
Thermal correction to Enthalpy= 0.413689
Thermal correction to Gibbs Free Energy= 0.354104
Sum of electronic and zero-point Energies= -851.171906
Sum of electronic and thermal Energies= -851.156028
Sum of electronic and thermal Enthalpies= -851.155084
Sum of electronic and thermal Free Energies= -851.214669
Maximum Force 0.000003 0.000450 YES
RMS Force 0.000000 0.000300 YES
Maximum Displacement 0.000232 0.001800 YES
RMS Displacement 0.000050 0.001200 YES

12

#M062X/6-31G(d) freq(noraman) nopop guess=read geom=check

1	6	0	2.978671	0.125564	-0.496534
2	6	0	3.627369	0.137670	0.895336
3	6	0	2.628473	-0.393457	1.933504
4	6	0	2.236230	-1.828692	1.568837
5	6	0	1.582551	-1.846911	0.176876
6	6	0	2.569478	-1.307268	-0.868438
7	6	0	1.375139	0.495414	1.948939
8	6	0	0.318047	-0.970730	0.24289
9	6	0	0.698073	0.498227	0.557434
10	6	0	-0.515844	1.411980	0.501164
11	6	0	-1.715702	1.027551	-0.154095
12	6	0	-2.805151	1.926482	-0.215531
13	6	0	-2.719347	3.184373	0.345193
14	6	0	-1.539390	3.561561	0.990365
15	6	0	-0.460341	2.686561	1.063178
16	6	0	-1.823118	-0.273554	-0.771857
17	6	0	-0.571744	-1.017109	-1.057392
18	6	0	-0.728177	-2.364597	-1.643333
19	6	0	-1.942806	-2.902399	-1.907328
20	6	0	-3.107847	-2.142745	-1.600024
21	6	0	-3.056755	-0.871694	-1.060121
22	6	0	1.731912	1.019334	-0.476759
23	1	0	3.684472	0.511539	-1.239494
24	1	0	3.936991	1.157699	1.154072
25	1	0	4.531265	-0.482903	0.894014
26	1	0	3.084526	-0.377955	2.928946

27	1	0	1.543221	-2.240191	2.313094
28	1	0	3.122175	-2.474245	1.560189
29	1	0	1.302831	-2.880310	-0.055220
30	1	0	2.132086	-1.324865	-1.877767
31	1	0	3.452024	-1.956262	-0.901961
32	1	0	1.666267	1.513076	2.229059
33	1	0	0.659203	0.144119	2.703112
34	1	0	-0.317908	-1.346113	1.057265
35	1	0	-3.705413	1.650875	-0.753798
36	1	0	-3.552956	3.873952	0.273530
37	1	0	-1.457702	4.548231	1.435578
38	1	0	0.442478	3.013634	1.567403
39	1	0	-0.011030	-0.429595	-1.809822
40	1	0	0.179125	-2.909555	-1.887885
41	1	0	-2.044268	-3.889016	-2.344445
42	1	0	-4.082030	-2.586343	-1.789106
43	1	0	-3.983652	-0.369648	-0.810783
44	1	0	2.005136	2.044626	-0.198427
45	1	0	1.288996	1.090575	-1.480085

SCF Done: E(RM062X) = -851.601175774 A.U. after 1 cycles
Frequencies -- 55.1377 76.1251 103.7855
Zero-point correction= 0.400155 (Hartree/Particle)
Thermal correction to Energy= 0.415346
Thermal correction to Enthalpy= 0.416291
Thermal correction to Gibbs Free Energy= 0.359001
Sum of electronic and zero-point Energies= -851.201021
Sum of electronic and thermal Energies= -851.185829
Sum of electronic and thermal Enthalpies= -851.184885
Sum of electronic and thermal Free Energies= -851.242175
Maximum Force 0.000016 0.000450 YES
RMS Force 0.000002 0.000300 YES
Maximum Displacement 0.000954 0.001800 YES
RMS Displacement 0.000106 0.001200 YES

13(TS)

#M062X/6-31G(d) freq(noraman) nopop guess=read geom=check

1	6	0	2.520286	1.576683	1.433082
2	6	0	3.477702	1.953490	0.280095
3	6	0	2.765391	2.404884	-1.006815
4	6	0	1.775214	3.536489	-0.708530
5	6	0	0.760578	2.805688	0.189391
6	6	0	1.424315	2.633299	1.566678
7	6	0	1.809692	1.323692	-1.512385
8	6	0	0.568992	1.435476	-0.548751
9	6	0	0.629972	0.209315	0.338266
10	6	0	0.271894	-1.091868	-0.297636
11	6	0	-1.105733	-1.290602	-0.496668
12	6	0	-1.588362	-2.380318	-1.225168
13	6	0	-0.675795	-3.305613	-1.707849
14	6	0	0.695425	-3.142210	-1.469577
15	6	0	1.178692	-2.051565	-0.756543
16	6	0	-1.858971	-0.267754	0.200296
17	6	0	-1.144806	0.274168	1.323455
18	6	0	-1.659023	1.450361	1.951925
19	6	0	-2.782736	2.064034	1.453951
20	6	0	-3.473035	1.494861	0.360692
21	6	0	-3.035434	0.329339	-0.251215

22	6	0	1.828085	0.208534	1.276359
23	1	0	3.111424	1.525870	2.353056
24	1	0	4.145698	1.110527	0.061560
25	1	0	4.115509	2.774149	0.629780
26	1	0	3.507048	2.675434	-1.764072
27	1	0	1.310408	3.888483	-1.636014
28	1	0	2.225921	4.398207	-0.206982
29	1	0	-0.193069	3.333474	0.276038
30	1	0	0.716630	2.331738	2.343754
31	1	0	1.841915	3.594350	1.883418
32	1	0	2.257876	0.325170	-1.533627
33	1	0	1.473235	1.543593	-2.529991
34	1	0	-0.354031	1.427448	-1.133399
35	1	0	-2.655438	-2.524106	-1.364793
36	1	0	-1.024050	-4.173221	-2.258157
37	1	0	1.395202	-3.874298	-1.859096
38	1	0	2.247987	-1.936695	-0.611689
39	1	0	-0.674505	-0.444856	1.991488
40	1	0	-1.198149	1.818808	2.861910
41	1	0	-3.176595	2.957219	1.926900
42	1	0	-4.362974	1.990851	-0.015946
43	1	0	-3.553608	-0.068340	-1.118046
44	1	0	2.539257	-0.538841	0.900778
45	1	0	1.519878	-0.168276	2.258325

SCF Done: E(RM062X) = -851.561657798 A.U. after 1 cycles
Frequencies -- -299.3640 63.2814 79.1469
Zero-point correction= 0.397654 (Hartree/Particle)
Thermal correction to Energy= 0.413035
Thermal correction to Enthalpy= 0.413979
Thermal correction to Gibbs Free Energy= 0.356175
Sum of electronic and zero-point Energies= -851.164004
Sum of electronic and thermal Energies= -851.148623
Sum of electronic and thermal Enthalpies= -851.147679
Sum of electronic and thermal Free Energies= -851.205483
Maximum Force 0.000003 0.000450 YES
RMS Force 0.000001 0.000300 YES
Maximum Displacement 0.000438 0.001800 YES
RMS Displacement 0.000095 0.001200 YES

7dp 6-31+G(d,p)

#M062X/6-31+G(d,p) freq(noraman) nopop guess=read geom=check

1	6	0	3.156424	0.276686	-0.098698
2	6	0	3.688548	-0.042416	1.314044
3	6	0	2.719680	-0.888742	2.155522
4	6	0	2.367313	-2.180738	1.412586
5	6	0	1.593722	-1.626617	0.205756
6	6	0	2.591287	-0.985637	-0.765569
7	6	0	1.343730	-0.224717	2.262764
8	6	0	0.664151	-0.535310	0.844560
9	6	0	0.683133	0.782962	0.202395
10	6	0	-0.493675	1.579572	0.029565
11	6	0	-1.772161	1.008313	-0.257831
12	6	0	-2.900041	1.825522	-0.177363
13	6	0	-2.786342	3.177300	0.133665
14	6	0	-1.529938	3.762471	0.334113
15	6	0	-0.396186	2.978092	0.259737
16	6	0	-1.903343	-0.359861	-0.801684
17	6	0	-1.046755	-0.785818	-1.827839
18	6	0	-1.174692	-2.062518	-2.369121
19	6	0	-2.156402	-2.926393	-1.888040

20	6	0	-3.020837	-2.505186	-0.875306
21	6	0	-2.904921	-1.225729	-0.341908
22	6	0	2.038181	1.337328	-0.115928
23	1	0	3.982981	0.681712	-0.690393
24	1	0	3.929483	0.890156	1.838579
25	1	0	4.629340	-0.595034	1.210295
26	1	0	3.144940	-1.066615	3.148063
27	1	0	1.733055	-2.822596	2.033474
28	1	0	3.242847	-2.761689	1.107577
29	1	0	0.972047	-2.371387	-0.297746
30	1	0	2.109629	-0.724817	-1.717503
31	1	0	3.388123	-1.699831	-0.994214
32	1	0	1.383908	0.839443	2.517345
33	1	0	0.715420	-0.711786	3.013182
34	1	0	-0.347521	-0.902155	1.000261
35	1	0	-3.871479	1.407138	-0.421820
36	1	0	-3.680386	3.791022	0.184558
37	1	0	-1.446923	4.820999	0.553405
38	1	0	0.571007	3.427319	0.455561
39	1	0	-0.312993	-0.095044	-2.238104
40	1	0	-0.518692	-2.375268	-3.175383
41	1	0	-2.259179	-3.920832	-2.309630
42	1	0	-3.788120	-3.175576	-0.502296
43	1	0	-3.571487	-0.905222	0.454113
44	1	0	2.267343	2.156369	0.581124
45	1	0	1.967769	1.811680	-1.103651

SCF Done: E(RM062X) = -851.621864095 A.U. after 2 cycles
Frequencies -- 31.9719 50.6113 67.9457
Zero-point correction= 0.395341 (Hartree/Particle)
Thermal correction to Energy= 0.411868
Thermal correction to Enthalpy= 0.412812
Thermal correction to Gibbs Free Energy= 0.351121
Sum of electronic and zero-point Energies= -851.226523
Sum of electronic and thermal Energies= -851.209996
Sum of electronic and thermal Enthalpies= -851.209052
Sum of electronic and thermal Free Energies= -851.270743
Maximum Force 0.000003 0.000450 YES
RMS Force 0.000001 0.000300 YES
Maximum Displacement 0.002120 0.001800 NO
RMS Displacement 0.000390 0.001200 YES

11ts 6-31+G(d,p)

#M062X/6-31+G(d,p) freq(noraman) nopop guess=read geom=check

1	6	0	3.260688	-0.742299	-0.372469
2	6	0	3.813391	-0.727063	1.061019
3	6	0	2.764955	-1.270272	2.044680
4	6	0	2.413022	-2.718084	1.697205
5	6	0	1.751562	-2.630645	0.284125
6	6	0	2.784499	-2.153928	-0.747239
7	6	0	1.473141	-0.462275	1.967273
8	6	0	0.674547	-1.629988	0.486914
9	6	0	0.877890	-0.245000	0.336357
10	6	0	-0.338061	0.644585	0.312046
11	6	0	-1.533406	0.210992	-0.301034
12	6	0	-2.633072	1.077091	-0.310713
13	6	0	-2.557946	2.347578	0.250311
14	6	0	-1.366984	2.782131	0.827125
15	6	0	-0.267197	1.931967	0.851936
16	6	0	-1.665837	-1.127961	-0.944212
17	6	0	-0.785831	-1.535746	-1.958100
18	6	0	-0.887093	-2.809067	-2.518833
19	6	0	-1.872021	-3.690194	-2.075560

20	6	0	-2.766963	-3.285119	-1.084400
21	6	0	-2.666456	-2.013343	-0.523742
22	6	0	2.077709	0.224437	-0.485876
23	1	0	4.042060	-0.419818	-1.067026
24	1	0	4.103668	0.292284	1.340638
25	1	0	4.714878	-1.345769	1.125141
26	1	0	3.138627	-1.199139	3.072833
27	1	0	1.716275	-3.144269	2.426525
28	1	0	3.292026	-3.367400	1.647770
29	1	0	1.298515	-3.591469	0.020453
30	1	0	2.341197	-2.154926	-1.749568
31	1	0	3.623542	-2.856668	-0.761387
32	1	0	1.622084	0.607885	2.135389
33	1	0	0.690930	-0.794088	2.655032
34	1	0	-0.267482	-1.964347	0.918936
35	1	0	-3.550631	0.747609	-0.789305
36	1	0	-3.423385	3.001319	0.225768
37	1	0	-1.293567	3.775343	1.256472
38	1	0	0.655983	2.284537	1.306310
39	1	0	-0.044730	-0.836277	-2.337694
40	1	0	-0.209607	-3.103214	-3.314811
41	1	0	-1.954835	-4.679849	-2.512458
42	1	0	-3.545262	-3.961699	-0.746452
43	1	0	-3.357574	-1.706531	0.256976
44	1	0	2.355587	1.233284	-0.161009
45	1	0	1.740118	0.317116	-1.524688

SCF Done: E(RM062X) = -851.609088059 A.U. after 2 cycles
Frequencies -- -197.4909 51.8165 58.4002
Zero-point correction= 0.394865 (Hartree/Particle)
Thermal correction to Energy= 0.410814
Thermal correction to Enthalpy= 0.411759
Thermal correction to Gibbs Free Energy= 0.351991
Sum of electronic and zero-point Energies= -851.214223
Sum of electronic and thermal Energies= -851.198274
Sum of electronic and thermal Enthalpies= -851.197330
Sum of electronic and thermal Free Energies= -851.257097
Maximum Force 0.000021 0.000450 YES
RMS Force 0.000003 0.000300 YES
Maximum Displacement 0.001291 0.001800 YES
RMS Displacement 0.000221 0.001200 YES

12 6-31+G(d,p)

#M062X/6-31+G(d,p) freq(noraman) nopop guess=read geom=check

1	6	0	2.978093	0.127519	-0.497945
2	6	0	3.628867	0.140820	0.893307
3	6	0	2.631814	-0.392058	1.932966
4	6	0	2.240677	-1.828046	1.569043
5	6	0	1.584834	-1.846683	0.177722
6	6	0	2.570216	-1.306267	-0.869233
7	6	0	1.377430	0.495511	1.950127
8	6	0	0.318873	-0.972408	0.245246
9	6	0	0.697844	0.496952	0.559554
10	6	0	-0.516564	1.409650	0.504806
11	6	0	-1.715194	1.027081	-0.154502
12	6	0	-2.803474	1.928743	-0.220069
13	6	0	-2.718306	3.186725	0.342636
14	6	0	-1.540259	3.561370	0.995009
15	6	0	-0.462338	2.684178	1.070587
16	6	0	-1.823418	-0.275248	-0.772409
17	6	0	-0.573890	-1.022915	-1.051765
18	6	0	-0.731639	-2.370776	-1.636026
19	6	0	-1.947507	-2.904249	-1.908239
20	6	0	-3.112341	-2.140582	-1.606926
21	6	0	-3.058900	-0.869013	-1.066687

22	6	0	1.729763	1.019598	-0.477110
23	1	0	3.682484	0.514331	-1.241794
24	1	0	3.937219	1.161214	1.151750
25	1	0	4.533755	-0.478031	0.890735
26	1	0	3.089452	-0.375888	2.927626
27	1	0	1.549363	-2.240231	2.314453
28	1	0	3.127133	-2.472631	1.559518
29	1	0	1.307211	-2.880725	-0.052995
30	1	0	2.133059	-1.324386	-1.878353
31	1	0	3.453360	-1.954160	-0.903459
32	1	0	1.668643	1.513022	2.229284
33	1	0	0.662967	0.143817	2.705680
34	1	0	-0.314649	-1.348833	1.061776
35	1	0	-3.702825	1.655736	-0.760823
36	1	0	-3.550366	3.877727	0.267592
37	1	0	-1.459270	4.546934	1.442655
38	1	0	0.438343	3.009986	1.579098
39	1	0	-0.014929	-0.439180	-1.809421
40	1	0	0.173951	-2.921167	-1.874607
41	1	0	-2.048831	-3.890672	-2.345686
42	1	0	-4.087203	-2.580755	-1.800667
43	1	0	-3.985260	-0.363510	-0.823050
44	1	0	2.002062	2.045227	-0.199969
45	1	0	1.285308	1.089156	-1.479658

SCF Done: E(RM062X) = -851.638547412 A.U. after 2 cycles
Frequencies -- 53.5019 74.7205 103.7069
Zero-point correction= 0.398001 (Hartree/Particle)
Thermal correction to Energy= 0.413262
Thermal correction to Enthalpy= 0.414207
Thermal correction to Gibbs Free Energy= 0.356769
Sum of electronic and zero-point Energies= -851.240546
Sum of electronic and thermal Energies= -851.225285
Sum of electronic and thermal Enthalpies= -851.224341
Sum of electronic and thermal Free Energies= -851.281779
Maximum Force 0.000019 0.000450 YES
RMS Force 0.000003 0.000300 YES
Maximum Displacement 0.001515 0.001800 YES
RMS Displacement 0.000322 0.001200 YES

13(TS) 6-31+G(d,p)

10-Mar-2025

****

#M062X/6-31+G(d,p) freq(noraman) nopop guess=read geom=check

1	6	0	2.522519	1.575262	1.433629
2	6	0	3.480366	1.948188	0.279126
3	6	0	2.768421	2.402765	-1.007661
4	6	0	1.781218	3.537329	-0.709002
5	6	0	0.765597	2.808767	0.190426
6	6	0	1.430286	2.635869	1.567888
7	6	0	1.809434	1.324148	-1.513172
8	6	0	0.570450	1.438547	-0.547469
9	6	0	0.625744	0.212299	0.342374
10	6	0	0.270794	-1.089045	-0.297282
11	6	0	-1.106445	-1.289077	-0.500007
12	6	0	-1.587364	-2.379846	-1.230199
13	6	0	-0.672649	-3.305005	-1.711818
14	6	0	0.698659	-3.140841	-1.469230
15	6	0	1.179514	-2.049126	-0.754057

16	6	0	-1.861983	-0.267402	0.196926
17	6	0	-1.144416	0.278459	1.316595
18	6	0	-1.660023	1.454780	1.945814
19	6	0	-2.791575	2.061103	1.455379
20	6	0	-3.486685	1.486019	0.366925
21	6	0	-3.045266	0.322627	-0.248186
22	6	0	1.824548	0.209449	1.280107
23	1	0	3.114985	1.523740	2.352686
24	1	0	4.144319	1.102510	0.059392
25	1	0	4.122134	2.765966	0.627887
26	1	0	3.510824	2.671747	-1.764804
27	1	0	1.316932	3.889971	-1.636543
28	1	0	2.233831	4.398356	-0.208359
29	1	0	-0.186268	3.340072	0.276655
30	1	0	0.723842	2.337947	2.347152
31	1	0	1.850945	3.596014	1.882649
32	1	0	2.255936	0.325310	-1.535547
33	1	0	1.472879	1.545269	-2.530418
34	1	0	-0.353014	1.434130	-1.131873
35	1	0	-2.653799	-2.525061	-1.372620
36	1	0	-1.018813	-4.172642	-2.263286
37	1	0	1.400021	-3.872429	-1.856781
38	1	0	2.248332	-1.934935	-0.606461
39	1	0	-0.674613	-0.439242	1.986572
40	1	0	-1.193907	1.829057	2.850619
41	1	0	-3.186777	2.952846	1.929899
42	1	0	-4.382594	1.975868	-0.003605
43	1	0	-3.567168	-0.078364	-1.111175
44	1	0	2.532172	-0.541099	0.905613
45	1	0	1.516571	-0.163229	2.263488

SCF Done: E(RM062X) = -851.599952217   A.U. after   2 cycles
Frequencies --   -306.5896   62.5308   78.1047
Thermal correction to Energy=   0.410974
Thermal correction to Enthalpy=   0.411919
Thermal correction to Gibbs Free Energy=   0.353966
Sum of electronic and zero-point Energies=   -851.204434
Sum of electronic and thermal Energies=   -851.188978
Sum of electronic and thermal Enthalpies=   -851.188034
Sum of electronic and thermal Free Energies=   -851.245986
Maximum Force   0.000003   0.000450   YES
RMS   Force   0.000001   0.000300   YES
Maximum Displacement   0.000410   0.001800   YES
RMS   Displacement   0.000079   0.001200   YES
